# CDK9 Inhibition with enitociclib reveals influence on HERV and LINE RNA abundances in whole blood, T-, and B-Cell lines

**DOI:** 10.1186/s12920-026-02309-6

**Published:** 2026-01-13

**Authors:** Nicholas Dopkins, Stephanie Michael, Nicholas Liotta, Fryda Solis Roldan, Douglas F. Nixon

**Affiliations:** 1 Northwell Health, New Hyde Park, NY United States; 2 Feinstein Institutes for Medical Research, Manhasset, NY United States; 3 Institute of Translational Research, FIMR, Manhasset, NY United States; 4 Zucker School of Medicine at Hofstra/Northwell-Hofstra University, Hempstead, NY United States; 5 Division of Infectious Diseases, Weill Cornell Medicine, New York, NY United States

**Keywords:** Human endogenous retrovirus (HERV), Endogenous retroelement (ERE), Long interspersed nuclear element (LINE), Lymphoma, CDK9, Positive transcription elongation factor b (P-TEFb)

## Abstract

Endogenous retroelements (EREs) comprise a significant portion of the human genome and there is a growing appreciation for their roles in eukaryotic physiology. In lymphomas, EREs may contribute to proto-oncogene expression and their RNA expression can be useful to better define lymphoma sub-classifications. Several emerging cancer therapies suggest that targeting ERE expression may even be beneficial to patient outcome. In this study, we investigated how enitociclib, a CDK9 inhibitor, impacted ERE expression over time. Using in vitro models of T and B lymphocytes, we found that CDK9 inhibition upregulates transcriptional abundances of ERE RNAs in a cell type- and temporal- specific manner. Leveraging this data with data collected from a retrospective cohort of patients with lymphomas receiving enitociclib, we found that ERE activity was initially downregulated, followed by a substantial upregulation in expression before a return to baseline expression levels within forty-eight hours. These results showed that ERE activity is differentially sensitive to CDK9 inhibition and highlights the complexity of interactions between the P-TEFb complex and nascent ERE RNAs.

**Supplementary Information:**

The online version contains supplementary material available at 10.1186/s12920-026-02309-6.

## Introduction

Human endogenous retroviruses (HERVs) and long interspersed nuclear elements (LINEs) are highly repetitive endogenous retroelements (EREs), loci derived from reverse transcription and integration, in the human genome [1–3]. HERVs and LINEs possess unique noncoding and coding functions which may greatly impact human health [1, 2, 4]. ERE RNA transcripts are substantially less abundant than those of canonical coding genes due to their extensive epigenetic repression [5, 6], however ERE RNAs are detectable in discrete spatiotemporal patterns throughout healthy somatic cells where they likely participate in physiological processes through poorly understood mechanisms [7–11]. Disease associated derepression of ERE expression is suggested to reinforce pathologies of aging [12–15], neurodegeneration [16–19], autoimmunity [20–22], and oncogenesis [23–25]. Loss of transcriptional regulation is a recurrently observed hallmark of hematological malignancies [26, 27] and this loss of regulatory control in cancerous cells may allow for the derepression of multiple EREs, therefore yielding unique expression profiles in certain cancers [25, 28–31]. Furthermore, EREs may encode tumor specific antigens [32–37], provide therapeutic targets [23], or function as circulating biomarkers [24]. Therefore, their continued study may likely improve upon detection and treatment strategies for a broad array of human cancers [38, 39].

Non-Hodgkin Lymphomas (NHLs) are a the classification of lymphocyte-derived cancers absent of the morphologically distinct Reed-Sternberg cells [40]. Diffuse large B cell lymphomas (DLBCLs) are a highly heterogenous subclassification of NHLs that account for ~ 150,000 new diagnoses annually [41]. DLBCLs primarily arise from malignant B cells that may form in the periphery or the germinal center [41]. The tumor microenvironment (TME) of B cell lymphomas represent a complex and heterogenous niche depending on the site, stage, individual, and mutagenic profile [42]. However, the B cell lymphoma TME typically displays hallmarks of immune evasion, which aid the malignant cells to grow, escape immune recognition, and potentially harbor a resistance to the standard of care treatments [41, 43]. While > 60% of patients with DLBCL can be effectively cured with the standard of care treatment via a combination of rituximab, cyclophosphamide, doxorubicin, vincristine, and prednisone (R-CHOP), patients with refractory or relapsing DLBCL typically face worsened prognostic outcomes and require alternative treatments, such as chimeric antigen receptor T cell (CAR-T cell) therapy [41].

Rearrangement of the protooncogene *MYC*, which permits TME growth and immune evasion [44], is a recurrent hallmark of the cases with poor prognostic outcomes [45], and transgenic mice overexpressing *MYC* represent a murine model of spontaneous lymphomagenesis [46]. While numerous CAR-T cell therapies for NHLs have gained FDA approval as second-line therapies greatly efficacious in allowing R-CHOP-resistant individuals to achieve long-term complete remission [47–59], the lingering complications posed by non-responders and accessibility indicate the heightened need for improved or alternative intervention strategies [60–65].

Rapid cell cycle progression and associated processes are hallmarks of certain cancers [66], chemotherapeutic interventions which inhibit the necessary processes required to reach a checkpoint of cell division have been implemented to treat many malignancy types [67]. Since this direct suppression of cell cycle progression or indirect inhibition of the cellular processes required for cell cycle progression is an important aspect of chemotherapies [68], we sought to investigate how targeting cancer cell proliferation and oncogene expression might impact the transcriptional regulation of EREs, a complex process that, when derailed, can impact human health [5]. In health, EREs can partake in the processes of reproduction [69, 70], development [71], and immunity [72], while during disease conditions they likely reinforce multiple pathologies [1]. In cancers, their aberrant activity in the genome [73–75], transcriptome [25, 31], and proteome [24, 33–35] likely further reinforces or initiates oncogenesis, and therefore it is of importance to classify how their activity is defined in human malignancies, and how their activity responds to therapy.

For this purpose, we investigated how ERE expression is sensitive to an emerging chemotherapeutic for lymphoproliferative malignancy treatment in enitociclib [76–82] by quantifying their expression in whole blood RNA sequencing and in vitro models of T and B lymphocytes before comparing to coding gene expression. To do this, we investigated how enitociclib, a cyclin dependent kinase 9 (CDK9) inhibitor, impacts ERE expression in Jurkat and Ramos cell line models of T and B cells which make up a significant fraction of the circulating leukocytes [83], respectively. We then leveraged these results by investigating the impact of enitociclib expression on EREs in whole blood RNA-sequencing data collected from a cohort of patients with either DLBCL or MYC positive NHL [76]. Mechanistically, CDK9 heterodimerizes with cyclins to form the positive transcription elongation factor b (P-TEFb) complex [84]. During transcription, RNA polymerase II (Pol II) complexes pause at the proximal-promoter region of a gene [84]. At this site of paused Pol II activity, P-TEFb recruitment reinitiates transcription [84]. This process of transcription elongation is a key stage in the quality control and regulation of gene expression. As a chemotherapeutic strategy, CDK9 inhibition aims to suppress transcriptional elongation of highly expressed oncogenes which permit cancer cell proliferation [85]. Retroviruses such as human immunodeficiency virus (HIV) exploit p-TEFb activity in the host cell to hyperactivate proviral transcription through the formation of stem loops possessing trans-activation response (TAR) domains that activate the retroviral accessory protein *Tat* [86]. While HERVs do not encode functional homologues of HIV *Tat*, this process indicates undescribed mechanisms by which endogenous proviruses and host transcripts differentially enlist the p-TEFb complex. Therefore, undescribed mechanisms may striate the regulation of transcriptional elongation by retroelement-derived RNAs when contrasted with RNAs of canonical origin. As many EREs may be differentially expressed in association with the differential expression of proto-oncogenes, primarily assumed to be upregulated in cancerous cells that possess relaxed transcriptional regulatory machinery when compared to non-cancerous, we sought to investigate how inhibition of the P-TEFb complex alters their activity in a time dependent manner.

## Results

### Differential impact of CDK9 Inhibition on RNA transcript category abundances in Jurkat cells

**Fig. 1:**
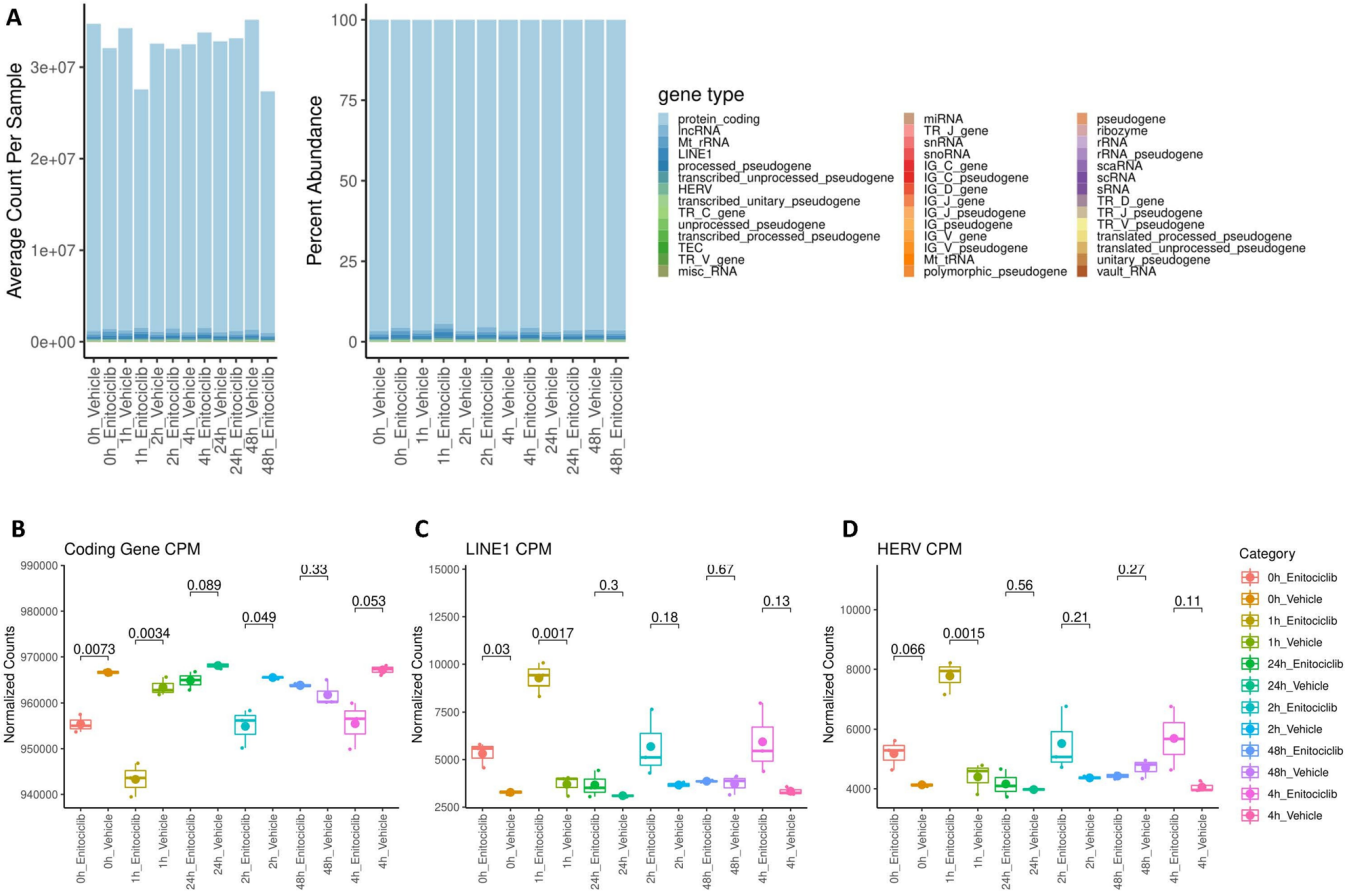
Differential impact of CDK9 inhibition on RNA transcript category abundances in Jurkat cells. The average count (left) of gene types sequenced per sample and the percent abundance (right) of gene types per sample sequenced from Jurkat cell lines receiving either vehicle or enitociclib treatment per each timepoint (**A**) (*n*=3 per group). The CPM abundance of protein coding genes transcripts (**B**), LINE transcripts (**C**), and HERV transcripts (**D**) from RNA sequencing of Jurkat cell lines receiving either vehicle or enitociclib treatment per each timepoint (*n*=3 per group). Tukey box and whisker plots represent all replicates with small dots, median expression with a midline, and mean with an enlarged dot. Displayed padj values for multiple comparisons were determined via Bonferroni corrected t testing for multiple comparisons. F test results accounting for variance between compared groups are available in Supplemental Table 11

In this study, we first investigated how a CDK9 inhibitor, enitociclib, influences ERE activity at the RNA level in cell line models of T and B lymphocytes. Characterization of RNA transcript types throughout the 48-hour timeline of Jurkat sequencing revealed that CDK9 inhibition fluctuates the counts and abundances of several RNA types (Fig. 1A; Supplemental Tabel 1). By considering transcript abundances in counts per million (CPM), we observed that protein coding genes are immediately downregulated upon exposure to enitociclib beginning at the 0-hour timepoint (Fig. 1B). This downregulation in protein coding gene abundance is conserved and statistically significant at 0-, 1-, and 2- hours before continuing as a near significant trend at 4- and 24- hours. By 48-hours, differences in protein coding gene abundances are indiscernible between vehicle and enitociclib treated Jurkat cells. Conversely, the CPM abundances of LINE (Fig. 1C) and HERV (Fig. 1D) transcripts display an immediate upregulation at the 0-hour time point that acutely persist at varying significances at 1-, 2-, and 4- hours post-treatment. This upregulation in ERE abundance then deescalated before being unobservable at the 24-hour and 48-hour timepoints. Collectively, these data demonstrate that T lymphocyte-derived cell line expression of HERVs and LINEs undergoes discrete transcriptional regulation en mass during CDK9 inhibition. Their acute upregulation suggests that EREs may evade or counter mechanisms of pTEF-b inhibition by relying on alternative cofactors for transcriptional elongation, or that their elevated expression may compensates for the repression of protein coding genes.

### Differential impact of CDK9 inhibition on RNA transcript category abundances in Ramos cells

**Fig. 2:**
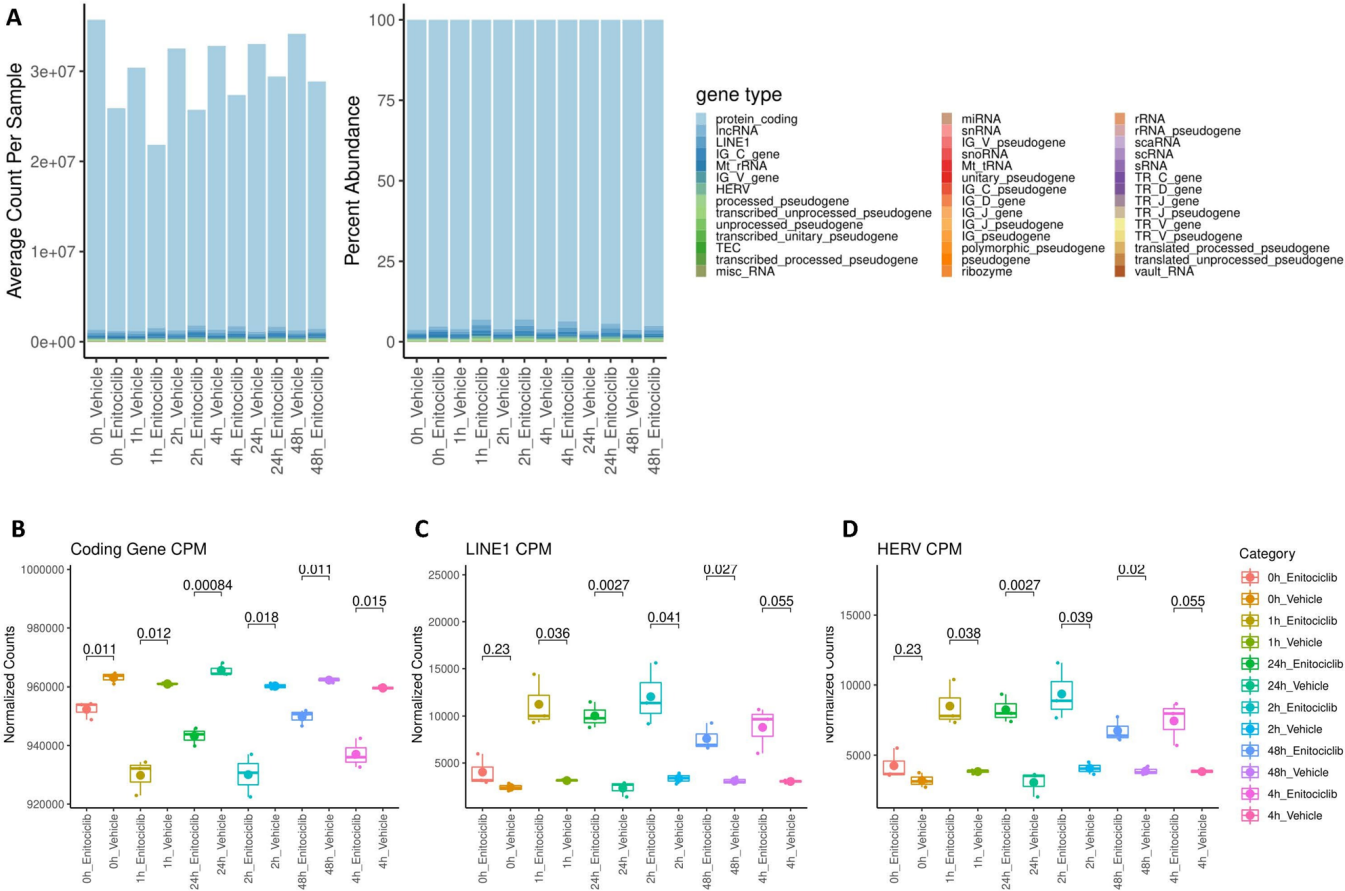
Differential impact of CDK9 inhibition on RNA transcript category abundances in Ramos cells. The average count (left) of gene types sequenced per sample and the percent abundance (right) of gene types per sample sequenced from Ramos cell lines receiving either vehicle or enitociclib treatment per each timepoint (**A**) (*n*=3 per group). The CPM abundance of protein coding genes transcripts (**B**), LINE transcripts (**C**), and HERV transcripts (**D**) from RNA sequencing of Ramos cell lines receiving either vehicle or enitociclib treatment per each timepoint (*n*=3 per group). Tukey box and whisker plots represent all replicates with small dots, median expression with a midline, and mean with an enlarged dot. Displayed padj values for multiple comparisons were determined via Bonferroni corrected t testing for multiple comparisons. F test results accounting for variance between compared groups are available in Supplemental Table 11

We next investigated the impact of enitociclib on ERE expression in a Ramos cell line model of B lymphocytes. Like Jurkat cells, the 48-hour timeline of Ramos sequencing revealed that CDK9 inhibition fluctuates the counts and abundances of several RNA types (Fig. 2A; Supplemental Table 2). By considering transcript abundances, we observe that protein coding genes are immediately downregulated upon exposure to enitociclib beginning at the 0-hour timepoint (Fig. 1B) and that this downregulation in protein coding gene abundance is conserved and statistically significant at every timepoint tested. In contrast to Jurkat cells, the CPM abundances of LINE (Fig. 1C) and HERV (Fig. 1D) transcripts do not upregulate following enitociclib treatment until the 1-hour timepoint. In further contrast to Jurkat cells, enitociclib-treated Ramos groups sustain significant upregulations in ERE transcript abundances at 1-, 2-, 24-, and 48- hours post-treatment with a near significant trend of upregulation at 4- hours. Collectively, these data demonstrate that B lymphocyte-derived cell line expression of HERVs and LINEs also undergoes discrete transcriptional regulation en masse during CDK9 inhibition. However, the elevated expression of ERE types in Ramos cell lines following enitociclib exposure is delayed and longer lasting when contrasted to Jurkat cells. This indicates cell type-specificity and temporal discrepancies in the upregulation of ERE transcript abundances following CDK9 inhibition

### Impact of CDK9 Inhibition on ERE expression in the Jurkat and Ramos cells

**Fig. 3:**
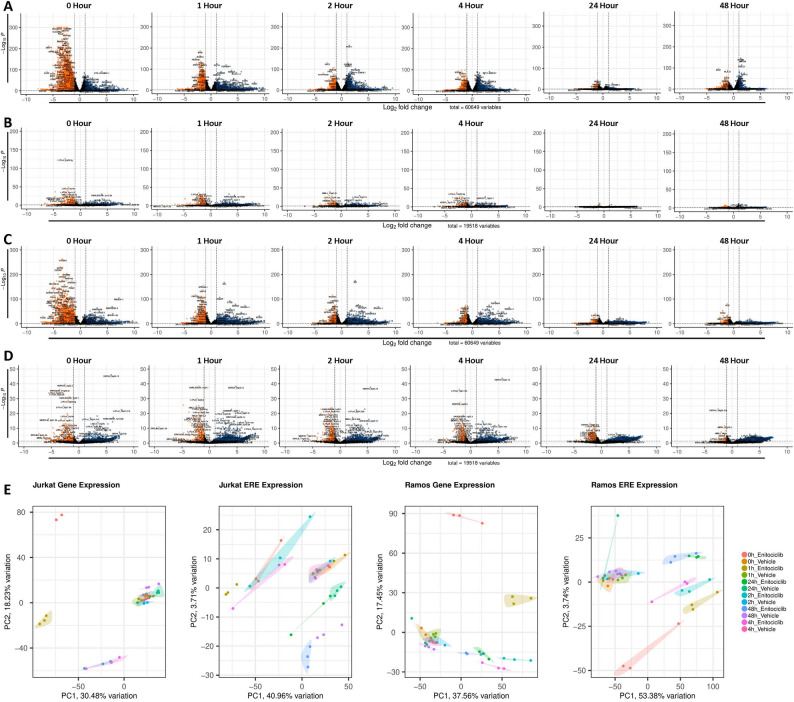
Impact of CDK9 inhibition on ERE expression in the Jurkat and Ramos cells. Differential expression of genes between vehicle controls and enitociclib treated Jurkat cell lines at 0- 1-, 2-, 4-, 24-, and 48-hours post treatment (**A**) (*n*=3 per group). Differential expression of EREs between vehicle controls and enitociclib treated Jurkat cell lines at 0- 1-, 2-, 4-, 24-, and 48-hours post treatment (**B** per group) (*n*=3). Differential expression of genes between vehicle controls and enitociclib treated Ramos cell lines at 0- 1-, 2-, 4-, 24-, and 48-hours post treatment (**C**) (*n*=3 per group). Differential expression of EREs between vehicle controls and enitociclib treated Ramos cell lines at 0- 1-, 2-, 4-, 24-, and 48-hours post treatment (**D**) (*n*=3 per group). Biplots of principal component (PC)1 and PC2 demonstrate clustering of samples by treatment condition and timepoint (**E**) (*n*=3 per group). Transcripts upregulated in enitociclib treated samples in comparison to vehicle controls are displayed in blue while those downregulated in enitociclib treated samples when compared to vehicle controls are displayed in red. Differential expression statistics were performed in DESEQ using the Wald's Test; adjusted *p* values were calculated using default parameters for a Benjamini-Hochberg correction. PCA plot shading represent a 95% confidence interval (CI). A total of 60,649 genes and 19,518 EREs were considered for differential expression analyses

We next investigated how the expression of individual ERE and protein coding gene loci are modulated in response to CDK9 inhibition (Supplemental Fig. S1). Immediately upon drug delivery, multiple protein coding gene loci are differentially expressed in Jurkat cells when compared to the vehicle treated group (Fig. 3A). The number of differentially expressed loci in enitociclib treated groups wanes by the 24-hour timepoint but remains observable at 48-hours. This indicates that the transcriptome of T lymphocyte cell line models possesses long-term susceptibility to CDK9 inhibition, yet the strongest impact can be observed at the acute stages of exposure. This contrasts with the differential expression of ERE loci, which are acutely deregulated by enitociclib exposure at the 0-, 1-, 2-, and 4- hour timepoints before better resembling expression patterns in the vehicle-treated group at 24- and 48-hours (Fig. 3B). In contrast, Ramos cells treated with enitociclib possess immediate differences in the transcriptional profiles of protein coding genes (Fig. 3C) and EREs (Fig. 3D) that persists at all tested time points.

Further investigation into the transcriptional abundances of 60 HERV families in vehicle- vs. enitociclib-treated Jurkat (Supplemental Fig. S2) and Ramos (Supplemental Fig. S3) cell lines demonstrate that individual HERV families primarily reflect the temporal trends observed in HERV CPM abundances (Figs. 1D and 2D), however numerous HERV families striate from these trends by being entirely unaffected or by being differentially expressed out of sync with the timepoint specific observations of ERE and HERV activity. This suggests that even within HERVs, there are likely factors that may discriminate their expression patterns following P-TEFb inhibition, potentially relating to their RNA structures or long terminal repeat (LTR) sequences or chromosomal location. Collectively, these results reveal the time course of protein coding gene and ERE activity following enitociclib treatment of T and B lymphocyte cell lines (Fig. 3E).

### Transcript category abundances in the whole blood of patients with lymphoma undergoing CDK9 Inhibition therapy

**Fig. 4:**
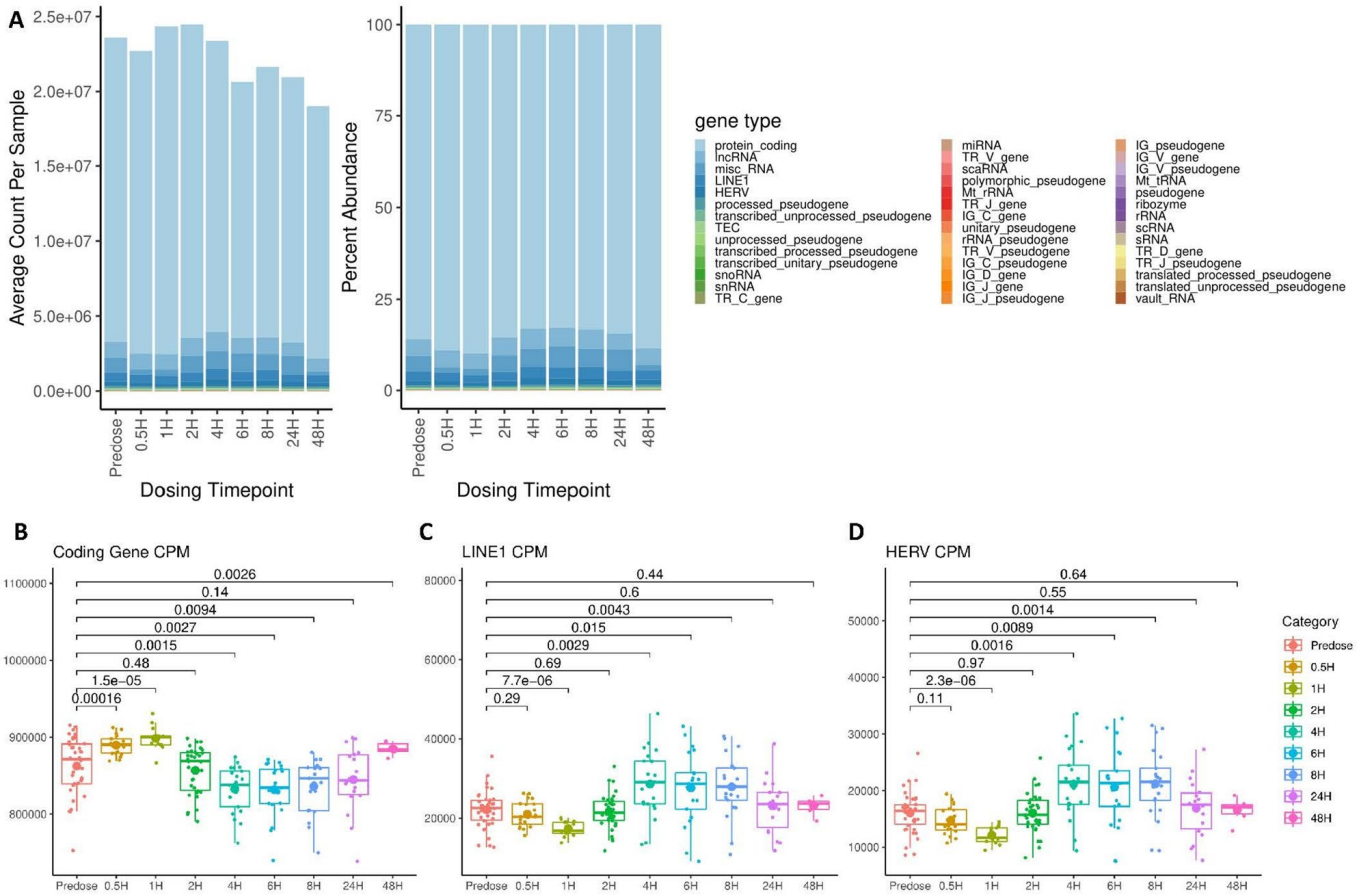
Transcript category abundances in the whole blood of patients with lymphoma undergoing CDK9 inhibition therapy. The average count (left) of gene types sequenced per sample and the percent abundance (right) of gene types per sample sequenced from whole blood of patients with lymphoma receiving enitociclib per each timepoint pre- and post-dosage (**A**) (predosage timepoint controls (*n*=38); 0.5 hours (*n*=20); 1 hour (*n*=13); 2 hours (*n*=34); 4 hours (*n*=21); 6 hours (*n*=21); 8 hours (*n*=21); 24 hours (*n*=18); and 48 hours (*n*=6)). The CPM abundance of coding genes transcripts (**B**), LINE (**C**) transcripts, and HERV (**D**) transcripts as identified in the Telescope annotation from sequencing performed on whole blood of patients with lymphoma receiving enitociclib per each time point pre- and post-dosage (predosage timepoint controls (*n*=38); 0.5 hours (*n*=20); 1 hour (*n*=13); 2 hours (*n*=34); 4 hours (*n*=21); 6 hours (*n*=21); 8 hours (*n*=21); 24 hours (*n*=18); and 48 hours (*n*=6)). Tukey box and whisker plots represent all replicates with small dots, median expression with a midline, and mean with an enlarged dot. Displayed adjusted *p* values for individual comparisons were determined via Bonferroni corrected t testing for multiple comparisons. Bartlett’s test results accounting for variance between compared groups are available in Supplemental Table 12

We next sought to describe how the chemotherapeutic application of enitociclib for patients with living with lymphoma influences the expression profiles of EREs in contrast with canonical coding genes. Characterization of RNA transcript types throughout the 48-hour timeline of whole blood sequencing revealed that CDK9 inhibition fluctuates the abundance and count of several RNA types (Fig. 4A; Supplemental Table 3). We observe that coding genes are initially upregulated at the 0.5-hour and 1-hour timepoints (Fig. 4B). However, their CPM abundance is then downregulated between the 2-hour to 24-hour time ranges. Conversely, the CPM abundances of LINE (Fig. 4C) and HERV (Fig. 4D) transcripts display a downregulation by the 1-hour timepoint that is then upregulated from the 4-hour to 8-hour timepoints. This upregulation in ERE abundance then deescalated before being unobservable at the 24-hour and 48-hour timepoints. Collectively, this suggests that HERVs and LINEs en masse undergo discrete transcriptional regulation during CDK9 inhibition to in vivo. While the 4-, 6-, and 8- hour timepoints reflect the general upregulation in ERE expression observed in T and B cell line models, nondifferential transcript abundances at 0.5- and 2-hours and the acute downregulation of ERE abundances in relation to protein coding genes at 1-hour indicate discrepancies between in vivo characterizations of whole blood and in vitro models of individual lymphocyte population. This can likely be explained by white blood cells making up a small percentage of circulating blood, and by temporal fluctuations of cell type abundances following drug treatment not testable with in vitro monocultures. Collectively, our analyses of primary samples and in vitro models suggest dynamic and c cell type-specific regulation of transcript type abundances by CDK9 inhibition, indicating that nuanced mechanisms determine pTEF-b efficacy at ERE loci when contrasted with those of protein coding genes.

### Impact of CDK9 Inhibition on ERE expression in the whole blood of patients with lymphoma

**Fig. 5:**
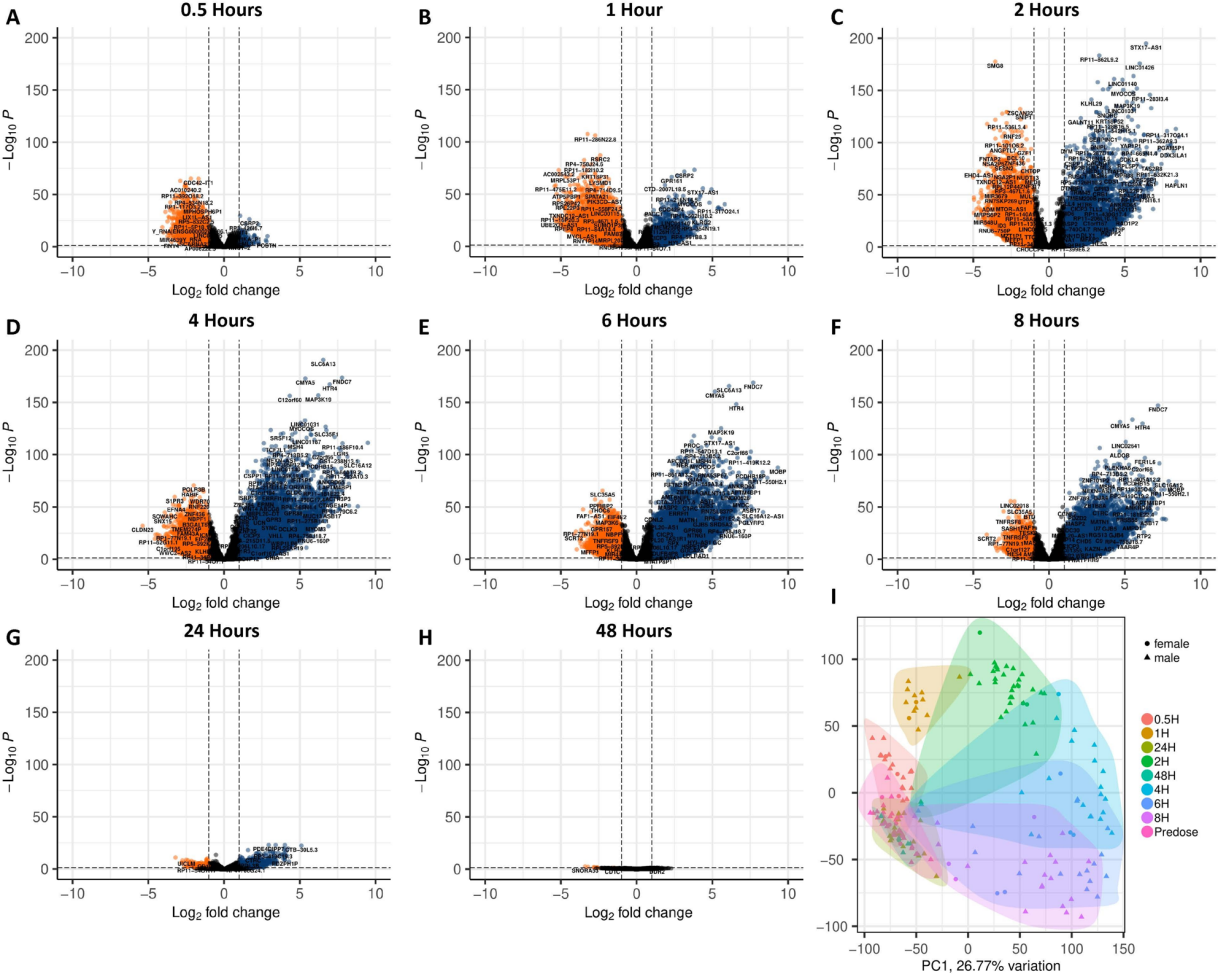
Impact of CDK9 inhibition on ERE expression in the whole blood of patients with lymphoma. Differential expression of EREs between predosage timepoint controls (*n*=38) and 0.5 hours (**A**) (*n*=20), 1 hour (**B**) (*n*=13), 2 hours (**C**) (*n*=34), 4 hours (**D**) (*n*=21), 6 hours (**E**) (*n*=21), 8 hours (**F**) (*n*=21), 24 hours (**G**) (*n*=18), and 48 hours (**H**) (*n*=6) post treatment. EREs upregulated in postdosage timepoints when compared to predosage controls are displayed in blue while those downregulated in postdosage timepoints when compared to predosage controls are displayed in red. Biplot of PC1 and PC2 demonstrate clustering of samples by timepoint based on ERE expression (**I**) (predosage timepoint controls (*n*=38); 0.5 hours (*n*=20); 1 hour (*n*=13); 2 hours (*n*=34); 4 hours (*n*=21); 6 hours (*n*=21); 8 hours (*n*=21); 24 hours (*n*=18); and 48 hours (*n*=6)). Differential expression statistics were performed in DESEQ using the Wald's Test; adjusted *p* values were calculated using default parameters for a Benjamini-Hochberg correction. PCA plot shading represent a 95% CI. A total of 13,808 EREs were considered for differential expression analysis following pre-processing filtering of EREs

We next investigated how ERE RNAs are modulated in whole blood following CDK9 inhibition at the locus level (Supplementary Fig. S4). At the 0.5-hour timepoint following enitociclib delivery, multiple ERE loci are substantially downregulated when compared to the predosage timepoint (Fig. 5A). At the one-hour and at the two-hour time points, ERE loci are increasingly up and down regulated, resulting in a large portion of these transcripts being differentially expressed (Fig. 5B-C). By the four-hour timepoint, the majority of differentially expressed ERE loci are upregulated (Fig. 5D), and this trend remains observable through the six and eight-hour post dosage timepoints (Fig. 5E-F). At twenty-four-hours post dosage, the activity of differentially expressed EREs restabilizes to more closely reflect their activity predosage (Fig. 5G), and by the forty-eight-hour time point only six EREs remain differentially expressed (Fig. 5H). Further investigation into 60 HERV families provided by the Telescope annotation demonstrates that individual HERV families undergo differential expression patterns in time dependent manners, with multiple being entirely unaffected or differentially expressed either in tune or out of sync with the general trend of ERE and HERV activity (Supplementary Fig. S5). This suggests that even within HERVs, there are likely factors that may discriminate their expression patterns following P-TEFb inhibition, potentially relating to their RNA structures or long terminal repeat (LTR) sequences. Collectively, these results show an acute time course of ERE activity following enitociclib treatment in the whole blood of lymphoma patients (Fig. 5I).

### Impact of CDK9 inhibition on canonical coding gene expression in the whole blood of patients with lymphoma

**Fig. 6:**
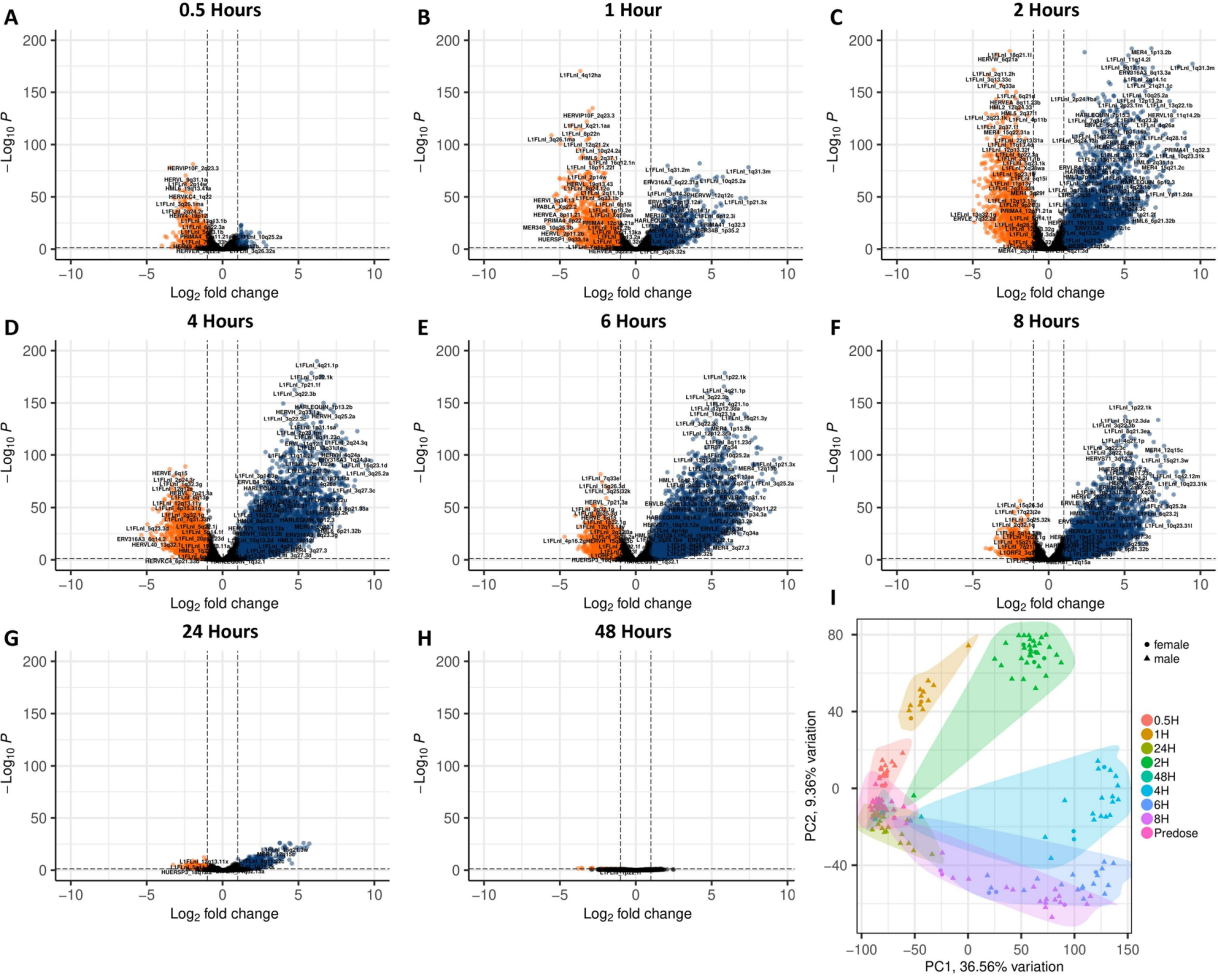
Impact of CDK9 inhibition on canonical coding gene expression in the whole blood of patients with lymphoma. Differential expression of coding genes between predosage timepoint controls (*n*=38) and 0.5 hours (**A**) (*n*=20), 1 hour (**B**) (*n*=13), 2 hours (**C**) (*n*=34), 4 hours (**D**) (*n*=21), 6 hours (**E**) (*n*=21), 8 hours (**F**) (*n*=21), 24 hours (**G**) (*n*=18), and 48 hours (**H**) (*n*=6) post treatment. Coding genes upregulated in postdosage timepoints when compared to predosage controls are displayed in blue while those downregulated in postdosage timepoints when compared to predosage controls are displayed in red. Biplot of PC1 and PC2 demonstrate clustering of samples by timepoint based on gene expression (**I**) (predosage timepoint controls (*n*=38); 0.5 hours (*n*=20); 1 hour (*n*=13); 2 hours (*n*=34); 4 hours (*n*=21); 6 hours (*n*=21); 8 hours (*n*=21); 24 hours (*n*=18); and 48 hours (*n*=6)). Differential expression statistics were performed in DESEQ using the Wald's Test; adjusted *p* values were calculated using default parameters for a Benjamini-Hochberg correction. PCA plot shading represent a 95% CI. A total of 60,649 genes differential were considered for differential expression analysis

We next considered if the patterns of ERE regulation at the locus level were reflected by similar patterns in coding gene expression. At the 0.5-hour timepoint following enitociclib delivery, multiple coding genes are substantially downregulated when compared to the predosage timepoint (Fig. 6A). At the one-hour and at the two-hour time points, coding genes are increasingly up and down regulated, resulting in a large portion of these transcripts being differentially expressed (Fig. 6B-C). By the four-hour timepoint, the majority of differentially expressed coding genes are upregulated (Fig. 6D), and this trend remains observable through the six and eight-hour post dosage timepoints (Fig. 6E-F). At twenty-four-hours post dosage, the activity of differentially expressed coding genes restabilizes to more closely reflect their activity predosage (Fig. 6G), and by the forty-eight-hour time point relatively few coding genes remain differentially expressed (Fig. 6H). Collectively, these results show an acute time course of coding gene activity following enitociclib treatment in the whole blood of lymphoma patients that reflects the cycle observed in EREs (Fig. 6I). Qualitative observations suggests that EREs and coding genes primarily are undergoing similar processes of transcriptional regulation, however the proportion and significance values of ERE loci tend to be more differentially expressed than that of coding genes.

## Discussion

We observed that the global landscape of RNA abundances are highly sensitive to CDK9 inhibition with enitociclib. Further analysis discovered that the repression of EREs, such as LINEs and HERVs, following enitociclib delivery significantly differed from that of coding genes. By considering RNA abundances, we first demonstrated that EREs are typically upregulated acutely by CDK9 inhibition in T cells and chronically in B cells in vitro. Next, we showed in vivo that EREs are initially downregulated at 1-hour before being largely upregulated from 4 through 8-hours. These trends suggest that EREs may undergo differing mechanisms of transcriptional elongation or depend upon unidentified cofactors that differ from those required by coding genes. Further investigation found that even among HERV families, there is largely heterogenous responses to CDK9 inhibition, allowing us to further hypothesize that the sequence identity of LTRs or coding regions of EREs may play a role in coordinating transcriptional elongation efficacy despite CDK9 inhibition. Mechanistically, whether HERVs functionally replicate TARs that interact with undescribed host- or ERE-derived factors similar to HIV *Tat* [86] is an intriguing possibility requiring further investigation. Future studies should emphasis the usage of reporter assays for *Tat* or LTR mimic constructs to identify the impact of endogenous regulators of HERV transcription through TAR formation. Gene ontology (GO) enrichment analysis of gene expression in Jurkat and Ramos cells are provided in Supplemental Tables 4 and Supplemental Table 5, respectively, which indicate numerous hypothetical mechanisms of transcriptional regulation and gene silencing based on reconstruction of the transcriptome for future mechanistic investigation.

This study possesses the following limitations. While cell line models suggest that changes in ERE RNA contents are due to an acute regulatory response, whether directly or indirectly, they do not account for the potential for chronic cumulative effects of enitociclib on transcript type abundances. In retrospective analyses of clinical cohorts, the gross effects are primarily ablated by the 48-hour timepoint, however this may be due to the low or fluctuating abundance of cell types exhibiting chronic cumulative effects. Furthermore, In vitro models of T and B lymphocytes represent a single genotype and do not reflect the potential impact of interindividual heterogeneity, environment, or other confounding factors when characterizing the impact of enitociclib treatment on protein coding gene and ERE expression profiles. Retrospective analysis of the clinical cohort of patients living with lymphoma and undergoing enitociclib treatment as a chemotherapy did not possess healthy controls, and therefore it can be difficult to determine if these changes are the result of CDK9 inhibition of all cells or result of CDK9 inhibition of malignant cells. The lack of healthy donors from the original cohort, and the utilization of pre-dosage controls limits the ability to consider the expression of EREs between individuals with and without lymphoproliferative malignancies. Additionally, the relatively small cohort size (*n* = 15) allows for unconsidered confounding factors to impact expression patterns. Temporal-specific changes in ERE activity in vivo are likely contributed to by fluctuations in cell type abundances present in whole blood, where the T and B lymphocytes analyzed with in vitro models represent a minute volume of the total cells. Additionally, not each patient provided samples for sequencing at each timepoint, and therefore the *n* value fluctuates throughout the examined time periods. Lastly, our bioinformatic workflow does not consider whether short interspersed nuclear elements (SINEs), another major subset of EREs, are sensitive to CDK9 inhibition. As LINE and HERV activity reverts to normality by the 48-hour timepoint, it is possible that SINE activity persists longer or is entirely unaffected. Based on these limitations, future studies that utilize single-cell RNA sequencing approaches or bulk RNA sequencing of fractionated cell populations derived from a larger cohort of healthy controls and patients living with lymphoma will better characterize the impact of CDK9 inhibition of ERE transcriptional abundances in vivo and will uncover which cell types of interest are responsible for specific striations from our in vitro findings. Future studies should emphasize immunoprecipitation-paired sequencing specific for Pol II, CDK9, or other facets of the P-TEFb complex to confirm the differential abundances of transcriptional regulatory machinery at ERE loci. This sequencing may be paired with immunoprecipitation-paired mass spectrometry specific for the same targets to determine whether differential or alternative components, cofactors, or modifications of the P-TEFb complex are responsible for differential ERE expression resulting from CDK9 inhibition.

Future research should assess how the P-TEFb complex interacts with different transcript types which possess distinct molecular characteristics, as this data raises hypotheses that unidentified cofactors or undescribed processes may influence transcriptional elongation at ERE loci. Further research is also required into how cancer therapeutics impact ERE activity, and whether this activity has biological implications to predict markers of therapeutic efficacy.

## Methods

### Cell culture and RNA extraction

Ramos and Jurkat cells lines were grown in complete RPMI (cRPMI) supplemented with 10% fetal bovine serum, 2mM L-glutamine, 100 units/mL of penicillin and 100 µg/mL of streptomycin. At day 0, Ramos and Jurkat cells were plated at 1 million live cells/mL, as determined by trypan blue staining at a final concentration of 0.04%, in 12 well plates and given either DMSO vehicle or 1µM enitociclib supplemented cRPMI. Cells were harvested at 0-, 1-, 2-, 4-, 24-, and 48- hours following exposure to vehicle or enitociclib supplemented media. Cells were harvested by centrifugation for 5 min at 300G. Supernatants were decanted prior to resuspension of cell pellets in Trizol reagent for lysis and storage at −20 °C. RNA contents were extracted from Trizol suspensions using a phase separation with chloroform prior to precipitation with isopropanol. RNA contents resuspended within isopropanol were further purified using RNeasy columns and washed using the RPE and RW1 buffers supplied according to manufacturer instructions provided by RNeasy kits. Following the elution of RNA contents from RNeasy columns, RNA contents were further purified using RNAClean XP beads according to manufacturer instructions. Purified RNA were then library prepped using a polyA selection kit for removal of rRNA and sequenced to an anticipated sequencing depth of 30 million reads per sample at a GeneWiz facility. All work with Jurkat and Ramos cell lines was performed 3 times as separate replicates. The Ramos cell line (clone RA-1) was provided by Ethel Cesarman and the Jurkat cell line (clone E6-1) was provided by Robert Furler O’Brien.

### Locus-specific quantification of ERE expression

RNA sequencing FASTQ of whole blood was obtained from a previously published study [76] under the bioproject accession number “PRJNA1021230”. FASTQ files generated by this study and obtained from PRJNA1021230 were aligned to the human genome build 38 (hg38) using STAR (v2.7.9.a) [87]. STAR alignment was performed using the parameters “--outSAMstrandField intronMotif --outFilterMultimapNmax 200 --winAnchorMultimapNmax 200” to retain multimapping ERE reads. We then used Telescope (v1.0.3) [88] to approximate the expression of HERVs and LINEs. The Telescope assign module was performed using the parameters “--theta_prior 200000 -- max_iter 1000” to align reads to an annotation of HERV and LINE sequences accessible at https://github.com/mlbendall/telescope_annotation_db. Metadata for the Telescope annotation can be found at https://github.com/liniguez/Telescope_MetaAnnotations. Stable gene ID count matrices are provided in Supplemental Tables 6 and Supplemental Table 7. Telescope count matrices are provided in Supplemental Tables 8 and Supplemental Table 9. Metadata for Jurkat and Ramos cell FASTQ files is provided in Supplemental Table 10. All supplemental tables and metadata annotations used or generated by this study for differential expression analysis are available at https://github.com/NicholasDopkins/Enitociclib.

### Differential expression analysis

Lowly abundant EREs were initially filtered by ensuring that all EREs considered for formal analyses possessed at least 2 reads within 10% of the total samples. Differential expression analysis was performed on ERE and protein coding gene expression profiles of whole blood collected at different dosing timepoints using DESEQ2 (v1.30.1) [89] with the parameters “parallel = T” and “betaPrior = T”. Differential expression analysis was performed on ERE and protein coding gene expression profiles of Jurkat and Ramos cells collected at different dosing timepoints following Enitociclib treatment DESEQ2. Results were extracted as DESEQ objects with a numbered contrast of each group compared against all others. Transcripts possessing a log2 fold change of > 1 or <−1 and an adjusted *p* value (padj) of < 0.05 when compared to their corresponding controls were assumed as differentially expressed. Visualization of transcript expression levels and differential expression were performed with the R packages pheatmap (v1.0.12) and EnhancedVolcano (v1.8.0) [90], respectively. Principal coordinate analyses (PCA) were performed on variance stabilizing transformed DESEQ outputs using PCAtools (v2.2.0) with the variable parameter set to “removeVar = 0.1”. ERE and protein coding transcript abundances were visualized with a custom function that overlays geom_jitter and geom_boxplot functions provided by ggplot2 (v3.3.5) [91] to produce a Tukey box and whisker plot displaying replicates, median, range, and mean.

### Gene ontology enrichment

GO enrichment analysis was performed on differentially expressed transcripts with a log2 fold change of > 1 or <−1 and a padj of < 0.05 when comparing timepoint controls to enitociclib treat Jurkat or Ramos cell lines using the R package topGO(v.2.42.0) [92].

### Statistics

The default Wald test within DESEQ2 [89] that provide a z score using shrunken estimates of log fold change divided by standard error and a Benjamini-Hochberg correction of *p* values was utilized to identify differentially expressed EREs and protein coding genes between the treated sample times and their vehicle or predosage controls. Adjusted *p* values were calculated to compare CPM abundances between time points by Bonferroni corrected t testing for multiple comparisons. Due to a lack of sample identifier information and variable n sizes per group, no paired statistical testing was utilized for the analysis of samples collected from PRJNA1021230. The equality of variances between 2 groups was performed using an F-test prior to unpaired t testing. The equality of variances between greater than 2 groups was performed using Bartlett’s test prior to ANOVA testing. Variance testing results are available within.

Supplemental Tables 11 and Supplemental Tables 12 and are available at https://github.com/NicholasDopkins/Enitociclib. GO term enrichment between vehicle and enitociclib treated cell line groups was calculated using Fisher’s exact test and are reported in Supplemental Tables 4 and Supplemental Table 5. Plotted padj values are represented to a definition of 2 significant digits. For data interpretation, all *p* values < 0.05 are referred to as significant.

### Ethics

Analysis of human subjects was performed on a retrospective cohort of individuals participating in the clinical trial “Phase I Dose Escalation Study for VIP152 in Patients With Advanced Cancer” (NCT02635672; Clinical trial registered on December 17th, 2015, and first posted on December 21 st, 2015). The authors had no impact on the study design, participant selection, or interpretation of initial results. All patient-derived data is publicly available and deidentified to remove personal identifiable information. Raw FASTQ files are catalogued and publicly available within the National Center for Biotechnology Information (NCBI) Sequence Read Archive (SRA).

## Supplementary Information

Supplementary Material 1.

Supplementary Material 2.

Supplementary Material 3.

Supplementary Material 4.

Supplementary Material 5.

Supplementary Material 6.

Supplementary Material 7.

Supplementary Material 8.

Supplementary Material 9.

Supplementary Material 10.

Supplementary Material 11.

Supplementary Material 12.

Supplementary Material 13.

## Data Availability

FASTQ files generated by this study are available at the NCBI SRA under bioproject accession number “PRJNA1271428”. FASTQ files gathered from a previously published study 76 are available at the NCBI SRA under bioproject accession number “PRJNA1021230”. Code for downstream analysis can be found at [https://github.com/NicholasDopkins/Enitociclib]**.**.
