# Supplementary material for: CDK9 Inhibition with enitociclib reveals influence on HERV and LINE RNA abundances in whole blood, T-, and B-Cell lines
Source: BMC Med Genomics. 2026 Jan 13;19:27. doi: 10.1186/s12920-026-02309-6 (PMC12888618; doi:10.1186/s12920-026-02309-6)
Supplement: Supplementary file 13 — Supplementary Material 13. [file 12920_2026_2309_MOESM13_ESM.pdf]

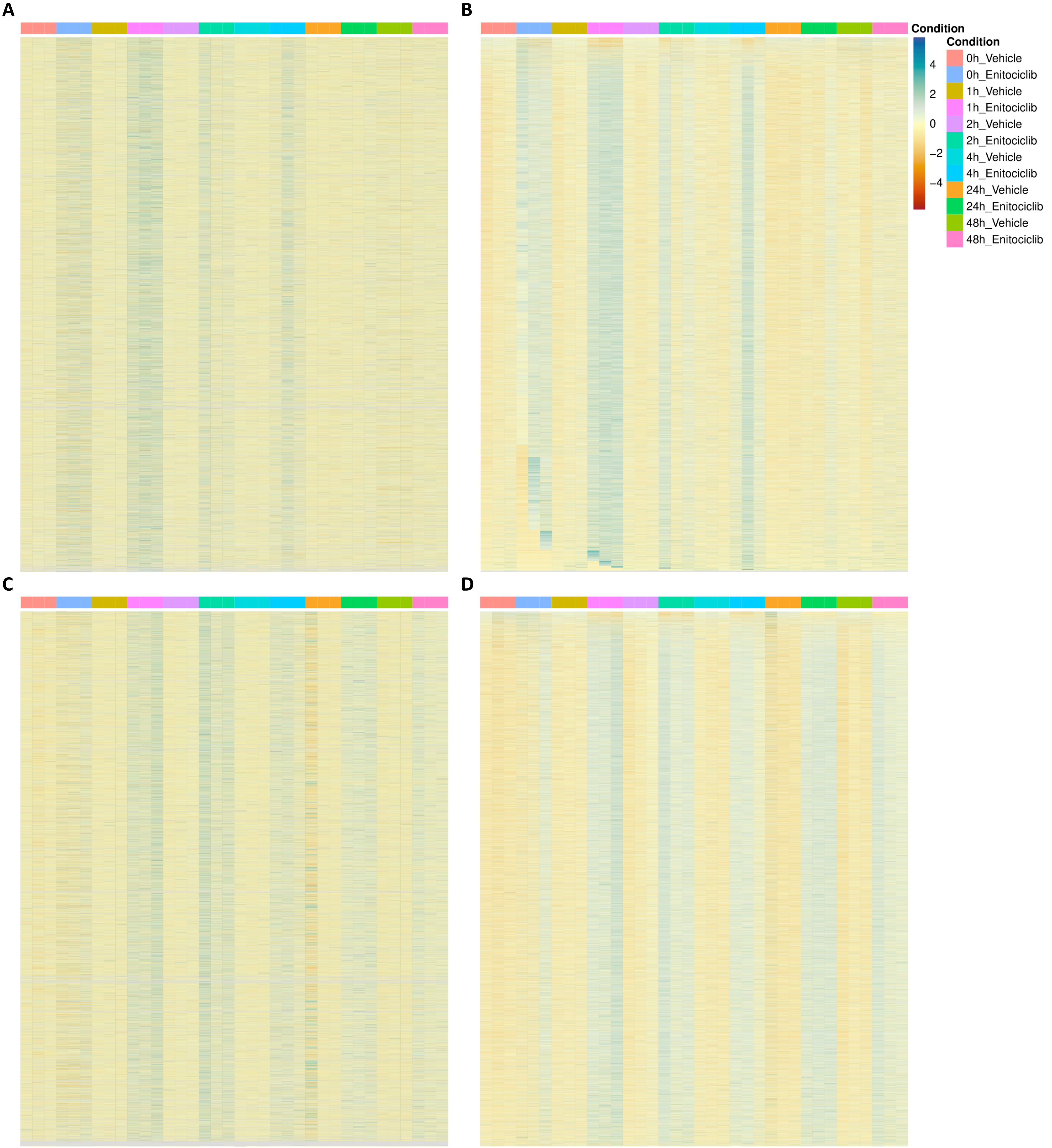

Supplemental Fig 1. Heatmaps of 60,649 genes and 19,518 EREs expressed in Jurkat cells and Ramos cells. Gene (A) and ERE (B) expression profiles in sequenced Jurkat cells undergone vehicle or enitociclib treatment at 0-, 1-, 2-, 4-, 24-, and 48- hours post treatment (n=3 per group). Gene (C) and ERE (D) expression profiles in sequenced Ramos cells undergone vehicle or enitociclib treatment at 0-, 1-, 2-, 4-, 24-, and 48- hours post treatment (n=3 per group).

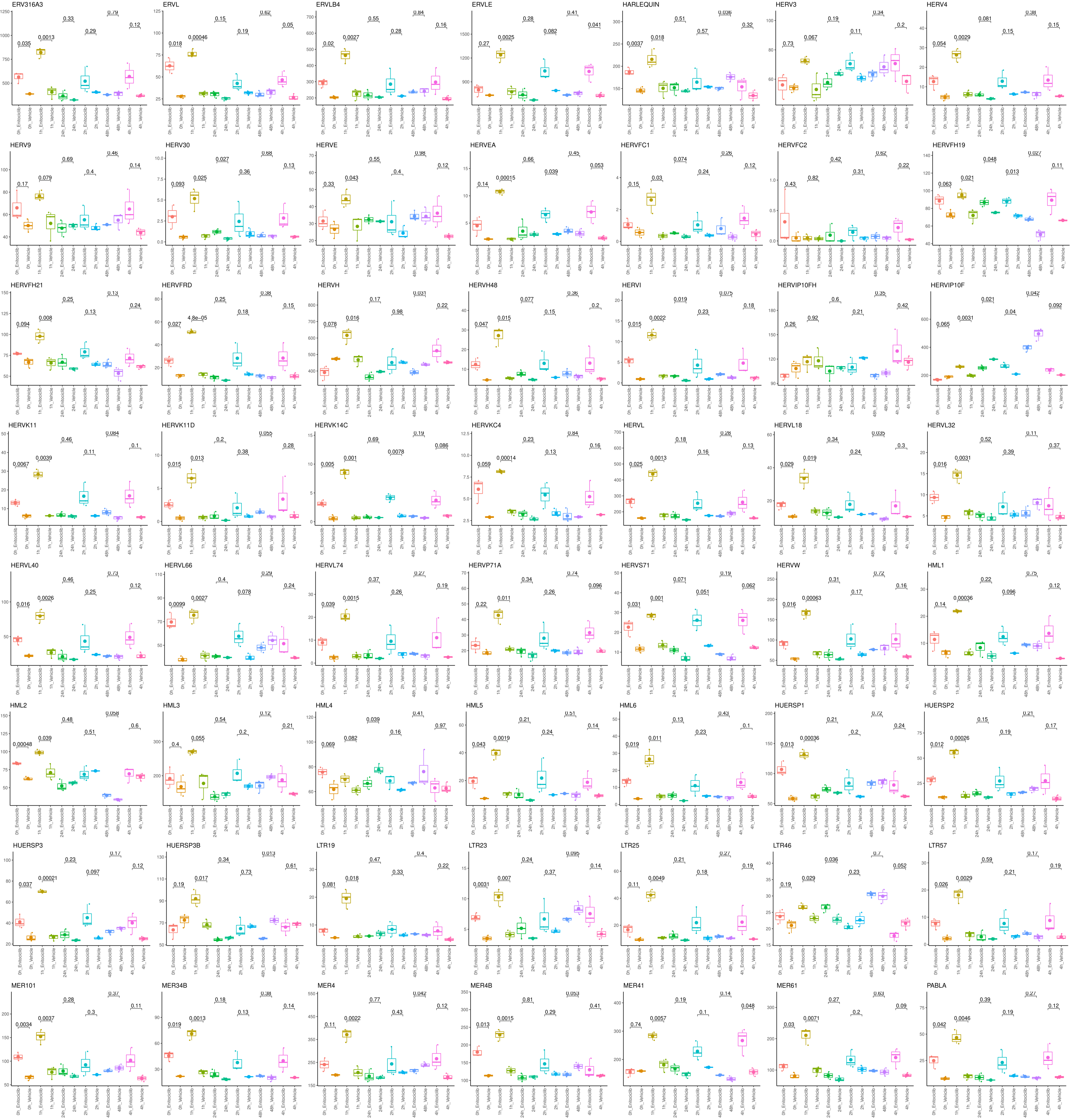
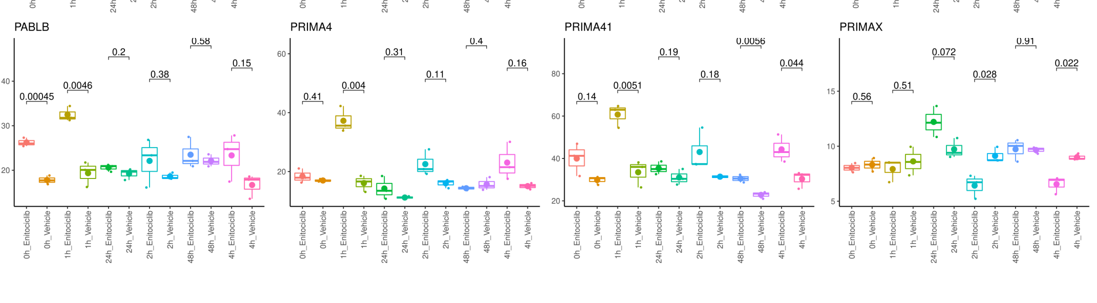

Supplemental Fig 2. HERV family level expression in sequenced Jurkat cells. CPM abundances of 60 HERV families annotated by the bioinformatic pipeline Telescope in sequenced Jurkat cells undergone vehicle or enitociclib treatment at 0-, 1-, 2-, 4-, 24-, and 48- hours post treatment (n=3 per group). Displayed padj values were calculated using multiple comparisons with a Bonferroni correction. F test results accounting for variance between compared groups are available in Supplemental Table 11.

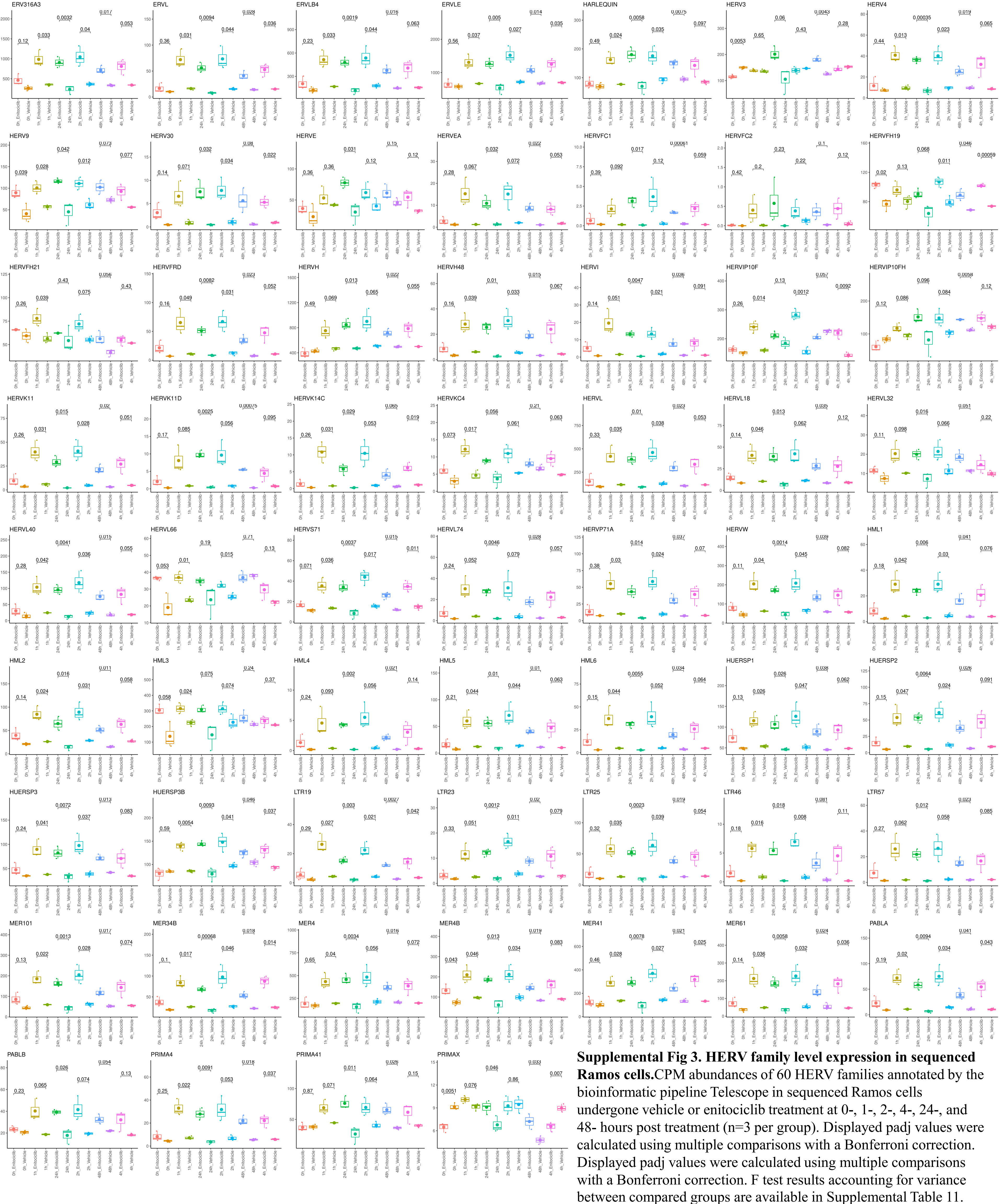

Supplemental Fig 3. HERV family level expression in sequenced Ramos cells. CPM abundances of 60 HERV families annotated by the bioinformatic pipeline Telescope in sequenced Ramos cells undergone vehicle or enitociclib treatment at 0-, 1-, 2-, 4-, 24-, and 48- hours post treatment (n=3 per group). Displayed padj values were calculated using multiple comparisons with a Bonferroni correction. Displayed padj values were calculated using multiple comparisons with a Bonferroni correction. F test results accounting for variance between compared groups are available in Supplemental Table 11.

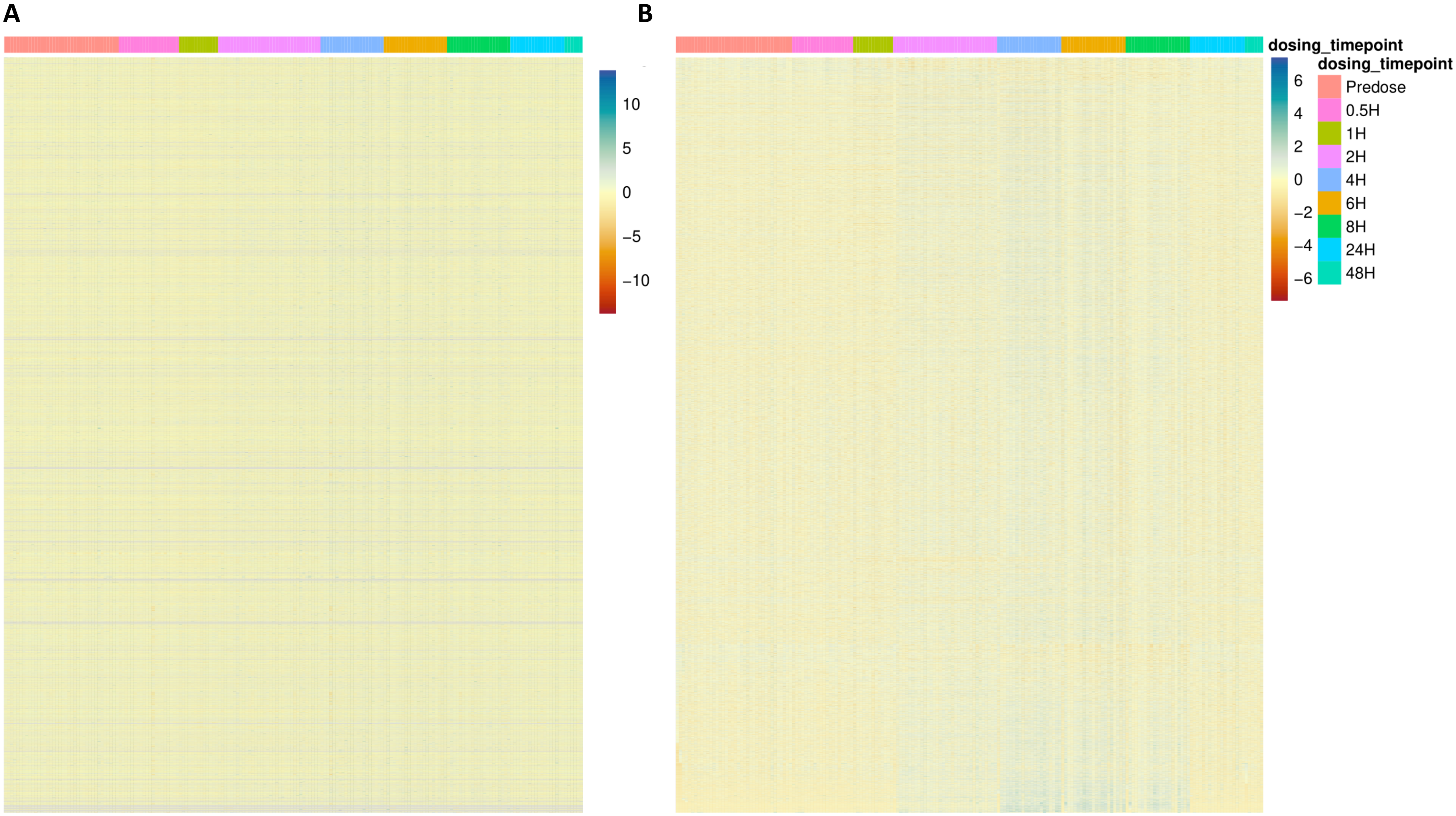

Supplemental Fig 4. Heatmaps of 60,649 genes and 13,808 EREs expressed in whole blood of patients living with lymphoma undergoing enitociclib treatment. Gene (A) and ERE (B) expression profiles in whole blood sequencing from patients living with lymphoma undergoing enitociclib treatment (predosage timepoint controls (n=38); 0.5 hours (n=20); 1 hour (n=13); 2 hours (n=34); 4 hours (n=21); 6 hours (n=21); 8 hours (n=21); 24 hours (n=18); and 48 hours (n=6)).

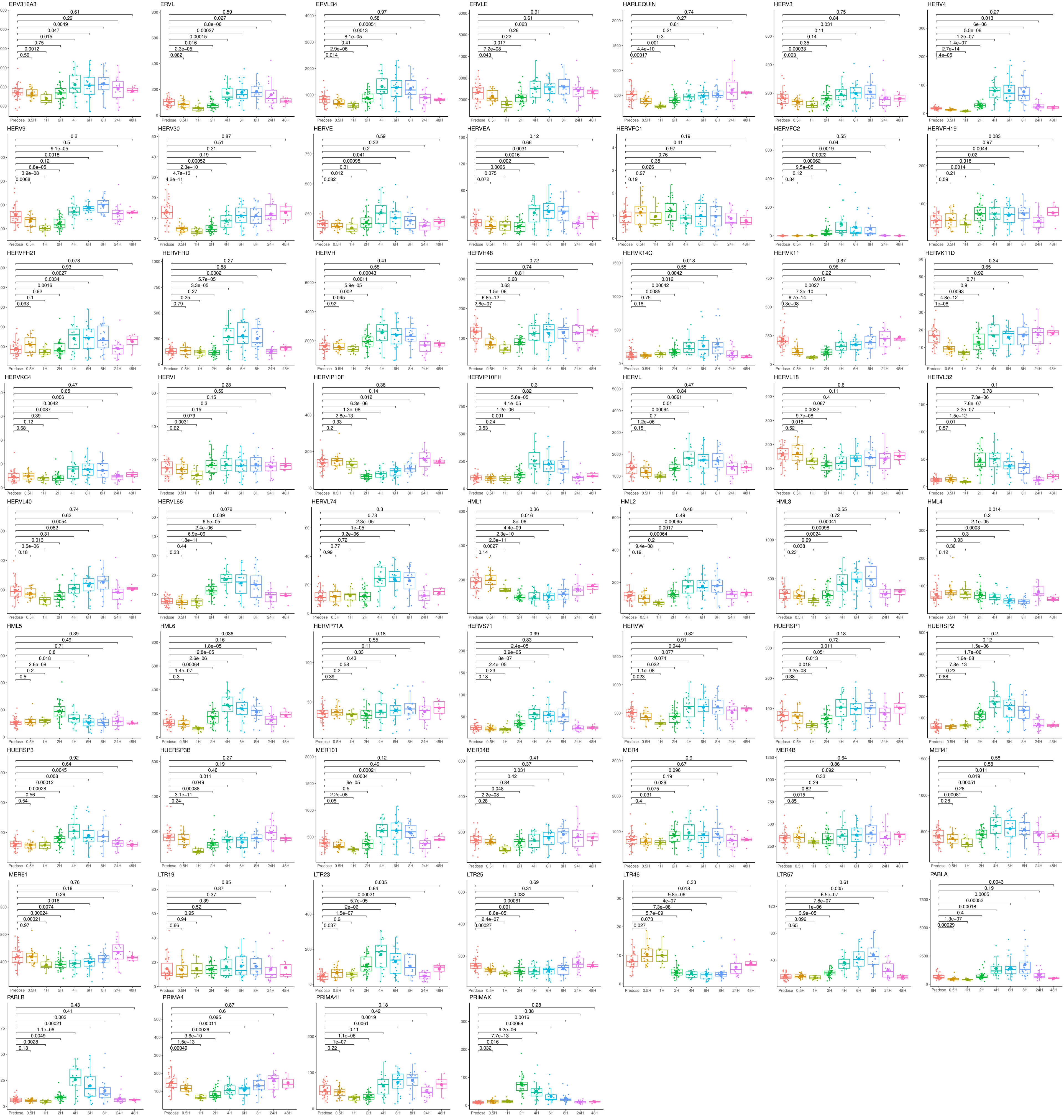

Supplemental Fig 5. HERV family level expression in whole blood of patients living with lymphoma undergoing enitociclib treatment. CPM abundances of 60 HERV families annotated by the bioinformatic pipeline Telescope in whole blood sequencing from patients living with lymphoma undergoing enitociclib treatment (predosage timepoint controls (n=38); 0.5 hours (n=20); 1 hour (n=13); 2 hours (n=34); 4 hours (n=21); 6 hours (n=21); 8 hours (n=21); 24 hours (n=18); and 48 hours (n=6)). Displayed padj values were calculated using multiple comparisons with a Bonferroni correction. Displayed padj values were calculated using multiple comparisons with a Bonferroni correction. Bartlett's test results accounting for variance between compared groups are available in Supplemental Table 12.
